# Ligament Injuries in Professional Male Handball Players: A 6-Year Longitudinal Study

**DOI:** 10.3390/healthcare12020201

**Published:** 2024-01-15

**Authors:** Luis Manuel Martínez-Aranda, Sergio García-Esteban, Manuel Sanz-Matesanz, Javier Raya-González

**Affiliations:** 1 Physical and Sports Performance Research Centre, Faculty of Sports Sciences, Pablo de Olavide University, 41013 Seville, Spain; 2 SEJ-680: Science-Based Training (SBT) Research Group, Pablo de Olavide University, 41013 Seville, Spain; 3 Faculty of Health Sciences, University Isabel I, 09003 Burgos, Spain; sergio.garcia.esteban@alumnos.ui1.es; 4 Club Balonmano Villa de Aranda, Medical Department, 09400 Burgos, Spain; 5 Faculty of Sport, Catholic University of Murcia, 30107 Murcia, Spain; msanz74@alu.ucam.edu; 6 Faculty of Sport Sciences, University of Extremadura, 06006 Cáceres, Spain; rayagonzalezjavier@gmail.com

**Keywords:** incidence, injury risk, team sport

## Abstract

Handball is a sport that involves high-intensity actions throughout the game, such as sprints, jumps, landings, and high-speed, repeated throws. This, along with competitive and tactical factors, congested schedules, and the need to maintain a high level of performance throughout the season, contributes to a high injury rate. This study aimed to analyse ligament injuries in a professional handball team over six consecutive seasons. A total of 68 elite male Spanish handball players participated, with 54 time-loss injuries (i.e., injuries involving at least one day of absence) observed during this study period. Ligament injury information was recorded following the International Olympic Committee consensus statement. The overall incidence was 0.89 ligament injuries per 1000 h of exposure. Additionally, a higher incidence and burden of ligament injuries was observed during match-play compared to training. Most ligament injuries were classified as minor or moderate (i.e., 79.63% of the total), and 46.29% were reinjuries. A significantly higher incidence of ligament injuries was suffered in the lower limbs compared to the upper limbs (0.81 vs. 0.08 ligament injuries per 1000 h; *p* < 0.001). Specifically, the highest incidence was observed in the anterior talofibular ligament of the ankle (0.57 injuries per 1000 h of exposure), while the greatest burden was related to the anterior cruciate ligament (24.08 absence days per 1000 h of exposure). This study provides an overview of ligament injuries among professional handball players, highlighting the need to implement strategies with positive effects during competition (e.g., specific activation strategies or training programmes based on strength and balance) and to reduce injury recurrences.

## 1. Introduction

Handball is a collaboration-opposition team sport which exposes players to substantial physical and physiological demands [1,2]. These demands include high-intensity actions like sprints, repetitive throws, and jumps with abrupt landings [3,4]. The permissive rules in professional handball, allowing player contact during the defensive phase [5], create situations prone to injuries [6]. The combination of these characteristics, along with congested schedules and the pressure on professional players to maintain a high level of competitiveness [7], has made handball the Olympic sport with the highest injury rate [8]. Injuries negatively impact team success [9], potentially causing traumatic (e.g., physical and psychological health issues) and long-term effects [10]. Additionally, injuries have financial implications for clubs due to high treatment costs and sick leaves [11], and players losing an entire season may lead to a loss of sponsorship funding [12]. Consequently, practitioners in handball have a compelling need to reduce the injury risk among their players.

To address this concern, a structured preventive approach must be implemented. Van Mechelen et al. [12] established epidemiological analysis as the first step in injury prevention because it facilitates the identification and description of injuries in terms of incidence and severity [12]. Many epidemiological studies focused on handball have been conducted [13,14,15]. However, a discrepancy in definitions used in previous epidemiological injury research makes it challenging to generalise findings [16]. In male elite handball players, injury incidence has been estimated between 4.1 and 12.4 injuries/1000 h of exposure [16,17], with higher rates during matches (8.3–14.3 injuries/1000 h) compared to training sessions (0.6–4.6 injuries/1000 h) [3,4]. A recent systematic review [18] determined that the ankle, knee, thigh, and lumbar regions have the highest injury incidence, with ligament sprains being the most common. However, specific studies analysing the incidence of ligament injuries are lacking. Also, previous studies are mainly focused on injury incidence or absolute values of severity (e.g., number of absence days), but without considering the associated burden (e.g., number of absence days/hours of practice).

Assessing professional athletes’ injury profiles requires considering more than just injury incidence due to its limitations. To gain a comprehensive understanding of the impact of injuries [19], it is essential to consider the number of absence days resulting from each injury. Severity, often presented in absolute terms, contributes significantly to the overall comprehension of injury episodes. However, to enhance the quality of these data, evaluating injury burden is advisable. This comprehensive concept combines the incidence rate, signifying the frequency of injuries, with a measurement of the extent of the loss incurred, providing a more insightful perspective [19]. Previous presentations of injury severity in handball [18] showed injuries with a duration of less than 7 days (i.e., 1–7 absence days) as most common, with a prevalence of slight injuries (i.e., 1–3 absence days) observed during international championships. However, only two epidemiological studies in handball players included burden values. Raya-González et al. [20] revealed values between 13.33 and 25.92 absence days/1000 h of training and between 1271.01 and 2594.10 absence days/1000 h of match in professional handball players. Raya-González et al. [21] obtained overall burden values of 46.19 absence days/1000 h of exposure among top male handball players, also providing burden values for training (14.88 absence days/1000 h of training) and matches (1436.20 absence days/1000 h of match). Knee and ankle sprain injuries appear most common in professional handball players [18], but the burden related to these injuries has not been longitudinally examined. Thus, incorporating injury burden into epidemiological analysis, along with specific information regarding different types of ligament injuries, is essential for designing and implementing individualised preventive training programmes. Moreover, it is crucial that this aligns with the predominant characteristics of ligament injuries experienced by handball players.

Given the limited literature specifically analysing ligament injuries in professional male handball players and the high incidence within this population, this study aims to examine the ligament injury profiles (e.g., incidence and burden) in a professional Spanish handball team over six consecutive seasons. The hypothesis posits high incidence and burden values during matches compared to training sessions, with the greatest burden associated with knee injuries, particularly those involving the anterior cruciate ligament (ACL). With this type of information, coaches and strength and conditioning specialists will be able to understand the importance and extent of this type of injury within this particular sport. Hence, they may focus their preventive training proposals for ligament injuries towards the maximum preservation and care of the structures involved in these injuries. In addition, the results expressed in this article will guide the mentioned professionals on which specific injury, location, and context may be the most interesting to address from the perspective of preventive training plan design.

## 2. Materials and Methods

### 2.1. Experimental Design

A prospective cohort design was employed to study ligament injury characteristics in professional male handball players over six consecutive seasons. The handball team remained in the top positions of the two major Spanish professional handball leagues for the complete evaluated period. All injuries during this period were recorded following the International Olympic Committee guidelines [22]. The club’s medical staff, consistent over the six seasons, were responsible for diagnosing, treating, and recording all ligament injuries across the entire period. Information on ligament injuries was recorded in an electronic database following the International Olympic Committee guidelines [22]. This information consisted of: absence as the player’s total number of absences in attendance (counted as days); severity as the injury’s classification based on related absence days divided into slight (1 to 3), minor (4 to 7), moderate (8 to 28), and major (more than 28); type, considering ligaments as the main focus of this research; location, being recorded as internal lateral ligament (elbow), internal lateral ligament (knee), anterior cruciate ligament (knee), or anterior talofibular ligament (ankle); and whether the injury occurred during training or match-play. The aforementioned information was used to establish differences between the types of ligament injuries recorded along this entire period.

### 2.2. Participants

Sixty-eight professional handball players (age = 26.7 ± 5.5 years, height 188.5 ± 15.5 cm; weight 87.8 ± 3.9 kg) from the same team in Spain participated in this study. A prior power analysis (G*Power, v3.1.9.2, Universität Kiel, Kiel, Germany) indicated that a sample size of at least 44 subjects was required to achieve power (1-β) of 0.90 with an effect size (ES) of 0.5 (large effect) and alpha of 0.05. The follow-up period spanned six consecutive seasons, with each player being part of the team for at least one season. Throughout the six seasons, the team conducted 6–7 in-court training sessions, 2–3 strength and conditioning training sessions, and 1–2 official matches per week. Players injured at the outset of this study were allowed to participate, but prior injuries were not recorded. All participants were informed of the investigation’s aim and participation was voluntary, with optional withdrawal at any time without consequences. The club officials’ assent was obtained, and all participants provided written consent. This study adhered to the Declaration of Helsinki, and the protocol was approved by the Ethical Committee of the University Isabel I, Spain (PUi1-008).

### 2.3. Definitions

Following the International Olympic Committee guidelines [22], an injury was defined as “a tissue damage or other derangement of normal physical function due to participation in sports, resulting from rapid or repetitive transfer of kinetic energy”, while a reinjury was defined as “subsequent injuries to the same location and tissue as the index injury if the index injury was healed/fully recovered”. Burden was presented as the number of absence days per 1000 h of exposure [19]. Exposure represented the time (in hours) during which players were exposed to injury risk in both training and match-play. Incidence referred to the number of injuries sustained during practice for every 1000 h of exposure. To calculate match-play exposure, only matches against teams from different clubs were considered, while training sessions involved physical activity directed by a team coach (on-court and strength and conditioning sessions). The club’s medical staff diagnosed, rehabilitated, and determined when an injured player was fully recovered.

### 2.4. Statistical Analysis

The Kolmogorov-Smirnov test ensured the normal distribution of data. Injury incidence (number of injuries/1000 h of exposure) and burden (number of absence days/1000 h of exposure) were calculated for each cohort with 95% confidence intervals (CI) [19]. Rate ratios (RR) with a 95% CI and the Z-test score [23] were calculated for injury incidence and burden to analyse differences between cohorts (e.g., training vs. matches or lower limbs vs. upper limbs). Statistical analyses were performed using a custom Microsoft Excel 2011 spreadsheet and GraphPad Prism v.6.0 c software, with a significance level set at *p* ≤ 0.05.

## 3. Results

Ligament injury incidence and burden during training, matches, and overall are presented in Table 1. A significantly greater ligament injury incidence was observed during matches (0.89 ligament injuries/1000 h of exposure) compared to training sessions (0.36 ligament injuries/1000 h of exposure). Similarly, a higher burden was reported during matches (1401.01 vs. 23.93 absence days/1000 h of exposure). No significant differences in incidence were observed regarding recurrence, although a higher burden was found for new injuries compared to reinjuries (5.02 vs. 30.12 absence days/1000 h of exposure).

Table 2 shows the differences in incidence and burden regarding severity. Minor and moderate injuries presented the greatest incidence (0.37 and 0.35 ligament injuries/1000 h of exposure, respectively). The highest burden was observed in major injuries (26.09 absence days/1000 h of exposure), followed by moderate (6.58 absence days/1000 h of exposure) and minor (2.33 absence days/1000 h of exposure) injuries.

Regarding ligament injury location, a significantly higher incidence of ligament injuries (0.81 vs. 0.08 injuries/1000 h of exposure) and burden (24.08 vs. 1.17 absence days/1000 h of exposure) were observed in the lower limbs compared to the upper limbs (*p* < 0.001). Differences in injury incidence and burden for each type of ligament injury are shown in Table 3. Ankle ATL was the most common type of ligament injury (0.65 ligament injuries/1000 h of exposure), while the highest burden was related to ACL injuries (24.08 absence days/1000 h of exposure).

## 4. Discussion

This study aimed to examine the profile of ligament injuries (e.g., incidence and burden) in a professional Spanish handball team over six consecutive seasons. It represents the first longitudinal investigation into the burden associated with ligament injuries in professional male handball players, including a specific analysis of the different types of ligament injuries. The results reveal an overall incidence of 0.89 ligament injuries per 1000 h of exposure. Furthermore, ligament injury incidence and burden were higher during match-play compared to training. The majority of ligament injuries were classified as minor or moderate (i.e., 79.63% of the total), and 46.29% were reinjuries. A significantly higher incidence of ligament injuries occurred in the lower limbs compared to the upper limbs (0.81 vs. 0.08 ligament injuries/1000h; *p* < 0.001). Specifically, the highest incidence was observed in the anterior talofibular ligament of the ankle (0.57 injuries/1000 h of exposure), while the greatest burden was associated with the anterior cruciate ligament (24.08 absence days/1000 h of exposure).

Injury incidence has been frequently used as a quantitative parameter to analyse the impact of injuries [24]. In this study, an overall incidence of 0.89 ligament injuries/1000 h of exposure was found, which is a lower value compared to overall injuries (i.e., between 4.1 and 12.4 injuries/1000 h exposure) [16,17]. However, this value is higher for a single type of injury, justifying the prevalence of ligament injuries in professional handball players [18]. Similar to most epidemiological studies with handball populations [3,4], the incidence related to matches is higher compared to that obtained during training sessions (23.93 vs. 0.36 ligament injuries/1000 h of exposure, respectively). These observed disparities may be attributed to a multitude of contributing factors. Primarily, the heightened physical and physiological demands placed on athletes during competitive matches, compared to training sessions, are crucial. The dynamic and unpredictable nature of handball introduces variability and uncertainty that significantly influences the physiological response of players during matches [25]. Moreover, the intricate interplay of neuromuscular and mental fatigue experienced during matches further exacerbates the divergence from training sessions.

In addition to these in-game factors, disparities may also arise from variations in the quantification and periodization of training loads [26]. Strength and conditioning coaches, given these multifaceted considerations, must adopt a comprehensive approach. This involves replicating not only the physical but also the technical, tactical, and psychological demands of competitive play during training sessions. Moreover, a key aspect is the strategic implementation of specific recovery protocols aimed at mitigating the detrimental effects of matches, such as accumulated fatigue and uncertainty [27]. By addressing these issues, coaches can effectively reduce the injury risk associated with handball practice, fostering the holistic development and resilience of their athletes. This approach aligns with the overarching goal of ensuring the well-being and optimal performance of handball players within the highly demanding and competitive sporting environment.

Despite the proven relevance of using incidence to quantify the impact of injuries, it is essential to also consider the consequences of these injuries in terms of their burden [19]. This holistic approach helps to express the meaningfulness of injury episodes. In our study, we revealed an overall burden of 33.64 absence days/1000 h of exposure. Similar to the incidence findings, this burden value appears lower compared to studies conducted by Raya-González et al. [20,21], which reported values close to 50 absence days/1000 h of exposure. This variance can be attributed to the inclusion of injuries with a higher associated burden, such as ACL injuries, in our study, as well as other less burdensome injuries, such as those resulting in 1 or 2 days of absence. On the other hand, the burden associated with match-related ligament injuries was significantly higher (i.e., 1401.01 absence days/1000 h of exposure) compared to the burden related to injuries sustained during training sessions (i.e., 3.02 absence days/1000 h of exposure). These discrepancies in burden between match and training-related ligament injuries can be attributed not only to differences in incidence but also to the specific types of injuries that occurred in each context. These findings underscore the importance of a comprehensive analysis, specifically evaluating the incidence and burden of various types of ligament injuries.

To address this concern, strength and conditioning coaches working with professional handball players may consider integrating strength training programmes into their periodization plans [28]. Such strength training programmes have demonstrated positive effects in reducing the burden associated with certain types of injuries as they help build stronger musculoskeletal structures, including muscles and joints. This, in turn, reduces the severity of injuries when athletes do get injured and facilitates their early return to play [29]. This approach also mitigates the detraining effects on physical and physiological variables, such as aerobic capacity, maximal oxygen consumption, blood composition (e.g., haematocrit), fat mass percentage, and lean mass percentage, which are negatively impacted by prolonged inactivity [30]. Furthermore, a reduced burden benefits the team’s performance since it results in shorter periods of absence and allows players to return under more favourable conditions, potentially achieving full participation sooner. It has been previously observed that the longer the period of absence, the more time it takes for an athlete to return to full match participation [29].

One of the primary objectives of reconditioning programmes is to ensure that the athlete returns to play in physical and physiological conditions that are either similar to or better than their state before the injury [31]. Another fundamental aim of these programmes is to reduce the risk of suffering a new injury. Previous studies have shown that a prior injury is the primary risk factor for sustaining a subsequent injury [32]. This is because a prior injury can lead to the formation of scar tissue within the musculature, creating less compliant areas with an increased risk of injury [33]. Additionally, a prior injury can result in changes along the kinematic chain, including proprioceptive deficits, reduced range of motion, excessive flexibility, and decreased strength, indirectly affecting the risk of injury [34]. Thus, the analysis of reinjuries is crucial for a better understanding of the problem. Surprisingly, in this study, no differences were observed between new injuries and reinjuries, even though the latter are typically less common in handball [18]. This might be attributed to extended downtime and the early return to play in an attempt to boost the team’s performance. In such cases, players may not be fully prepared to compete effectively and safely [29]. Nevertheless, the burden associated with reinjuries is lower when compared to new injuries, with relapses involving similar injuries but resulting in fewer days of absence. This could be explained by the nature/cause of the new injuries, as well as the recovery process and new preventative programme undertaken to address the immediately preceding injury. In essence, within this context and following the player’s complete recovery from an injury, the club’s fitness department immediately shifts focus to enhancing the condition of the structures involved in the mechanics of the previous injury. As a preventive measure, and regardless of the various reasons or factors that might contribute to a recurrence (e.g., direct physical impacts not due to overuse), such previous enhancement of the player’s fitness and the development of a robust muscular structure could provide a swifter and safer recovery compared to an injury in a new structure. This fact could facilitate a quicker return to play and minimise the associated burden of the recurrence.

In any case, given the high occurrence of reinjury, there appears to be a need to review the reconditioning processes, particularly when dealing with long-term injuries, as well as the criteria to return to play, ensuring that it is as safe and satisfactory as possible. Furthermore, the complete recovery of the athlete should take precedence over the team’s immediate sporting needs, as the consequences can be highly detrimental, especially in the case of long-term knee injuries [10].

Previous studies have indicated that injuries in professional male handball players primarily result in absences ranging from 1 to 7 days, accounting for approximately 65% of all injuries [35,36,37]. In contrast, our study found that minor injuries (i.e., 4 to 7 absence days) and moderate injuries (i.e., 8 to 28 absence days) were the most common severities, with incidence values of 0.37 and 0.35 ligament injuries per 1000 h of exposure, respectively. This pattern is reinforced by the nature of ligament injuries, which tend to be more severe than other types of injuries, contributing to an increase in their average severity. However, the greatest burden was observed in major injuries (i.e., 26.09 absence days/1000 h of exposure), which, although expected, is notably high, likely due to the nature of injuries sustained by these players, with some of them requiring more than 200 days of absence. As such, it becomes imperative to explore strategies aimed at reducing the burden associated with major injuries, thus preventing long-term injuries and addressing the issues linked to prolonged recovery periods. Regarding the location of ligament injuries, lower limb injuries exhibited a higher incidence (0.81 vs. 0.08 injuries/1000 h of exposure) and burden (24.08 vs. 1.17 absence days/1000 h of exposure) compared to upper limb injuries.

Despite the permissive rules governing handball [5], certain actions such as blocking and throwing are regulated, while pushing actions are more permissive and can lead to imbalances, making landings difficult and potentially contributing to the increase in lower limb injuries. Specifically, the most common type of ligament injury was the anterior talofibular ligament of the ankle, often attributed to actions such as twisting during lateral movements, landings, or accidental contact with teammates or opponents. In response to this trend, strength and conditioning coaches should consider implementing training programmes to enhance strength and create scenarios that mimic situations involving uncertainty or imbalances to better prepare players to handle such situations during practice. Conversely, the highest burden was associated with knee ACL injuries; although they are relatively infrequent, they result in significant time away from competition. As previously mentioned, ACL injuries carry severe consequences since players recovering from such injuries are not typically available for competitive play for up to 12 months post-surgical intervention [38]. Therefore, it seems necessary to develop strategies specifically focused on preventing ACL injuries, as even preventing a single occurrence of this injury can have a substantial positive impact, given its implications.

This study presents several limitations that practitioners should be aware of. Firstly, a case study approach, involving a single team, was employed in this study. This limitation makes it challenging to extrapolate the results to other populations, such as female or young handball players, primarily due to the differences in their epidemiological profiles when compared to male handball players [18]. However, this design allowed for a longitudinal study with a 6-year follow-up period, enabling the establishment of robust conclusions regarding the epidemiological analysis of ligament injuries in male professional handball players. Secondly, player availability in matches and training sessions was not recorded during the experimental period. This information would have been valuable in providing insights into the relationship between player availability and injury patterns. Future studies including this variable are needed to enhance the understanding related to this topic. Lastly, data related to training and match workload, as well as the fitness status of the players, were not collected during the study period. Consequently, the impact of fitness status and external and internal workloads on the injury profile of handball players could not be examined. Future research should consider these factors to provide a more comprehensive analysis of injury risk factors and prevention.

## 5. Conclusions

An overall incidence of 0.89 ligament injuries per 1000 h of exposure was obtained, with a higher incidence and burden of ligament injuries during match-play compared to training sessions. The majority of ligament injuries were classified as minor or moderate, while an astonishing 46.29% of all injuries were reinjuries. Moreover, lower limb injuries had a significantly higher incidence than upper limb injuries. The most commonly occurring ligament injury was related to the anterior talofibular ligament of the ankle, whereas the greatest burden was associated with anterior cruciate ligament injuries. This study provides a comprehensive overview of ligament injuries among professional handball players, underscoring the importance of implementing strategies that can have positive effects during competitive play. Such strategies may include specific activation techniques or strength and balance-based training programmes. Additionally, a focus on reducing reinjuries should be a priority for the welfare and performance optimisation of these athletes.

## Figures and Tables

**Table 1:** Ligament injury incidence and burden during training sessions, matches, and overall.

Injuries	Total (95% CI)	Training (95% CI)	Match (95% CI)	RR (95% CI)
Incidence	0.89 (0.69–1.17)	0.36 (0.23–0.55)	23.93 (17.01–33.66)	0.01 (0.03–0.28) ***
Burden	33.64 (32.20–35.14)	3.02 (2.61–3.50)	1401.01 (1339.91–1464.90)	0.01 (0.08–1.17) ***
Recurrence	Yes (95% CI)	No (95% CI)	RR (95% CI)	
Incidence	0.42 (0.28–0.62)	0.48 (0.34–0.70)	1.16 (0.68–1.98)	
Burden	5.02 (4.48–5.62)	30.12 (28.77–31.55)	6.01 (5.32–6.79) ***	

Abbreviations: Incidence = number of injuries/1000 h exposure; Burden = number of absence days/1000 h exposure; CI = confidence intervals. *** *p* < 0.001.

**Table 2:** Incidence and burden for each severity level of ligament injuries.

Injuries	Incidence (95% CI)	Burden (95% CI)
Slight	0.05 (0.02–0.15) ^a^***^,b^***	0.13 (0.07–0.27) ^a^***^,b^***^,c^***
Minor	0.37 (0.24–0.56) ^c^**	2.33 (1.98–2.75) ^b^***^,c^***
Moderate	0.35 (0.23–0.549) ^c^*	6.58 (5.96–7.26) ^c^***
Major	0.13 (0.07–0.27)	26.09 (24.83–27.42)

Abbreviations: Incidence = number of injuries/1000 h exposure; Burden = number of absence days/1000 h exposure; CI = confidence intervals. ^a^ = significant differences with minor; ^b^ = significant differences with moderate; ^c^ = significant differences with major; * *p* < 0.05; ** *p* < 0.01; *** *p* < 0.001.

**Table 3:** Injury incidence and burden in each type of ligament injury.

Injuries	Incidence (95% CI)	Burden (95% CI)
Elbow ILL	0.08 (0.03–0.20) ^c^*	1.16 (0.92–1.47) ^a^***^,b^***^,c^***
Knee ILL	0.15 (0.08–0.29) ^c^*	3.98 (3.51–4.52) ^b^***^,c^***
ACL	0.10 (0.04–0.22) ^c^*	24.08 (22.87–25.35) ^c^***
Ankle ATL	0.65 (0.47–0.89)	5.91 (5.33–6.56)

Abbreviations: Incidence = number of injuries/1000 h exposure; Burden = number of absence days/1000 h exposure; CI = confidence intervals; ILL = internal lateral ligament; ACL = anterior cruciate ligament; ATL = anterior talofibular ligament. ^a^ = significant differences with Knee ILL; ^b^ = significant differences with ACL; ^c^ = significant differences with Ankle ATL. * *p* < 0.05; *** *p* < 0.001.

## Data Availability

Data can be requested through the corresponding author.
